# Heterogeneity of cognitive aging in Brazilian normal elderls

**DOI:** 10.1590/S1980-57642009DN30400014

**Published:** 2009

**Authors:** Maria Paula Foss, Paulo Formigheri, José Geraldo Speciali

**Affiliations:** 1 Psychologist, PhD, Neurosciences Program, Department of Neurosciences and Behavioral Sciences, Faculty of Medicine of Ribeirão Preto, USP.; 2 Geriatrician, Master’s Degree, Program of Internal Medicine, Faculty of Medicine of Ribeirão Preto, USP.; 3 Associate Professor, Department of Neurosciences and Behavioral Sciences, Faculty of Medicine of Ribeirão Preto, USP.

**Keywords:** aging, neuropsychological tests, cluster analyses, cognition

## Abstract

With aging, several cognitive skills inevitably decline. However, cognitive losses do not occur homogenously in all elderly people, differing in number and severity of affected cognitive functions. These differences could be exacerbated by socioeconomic differences in a developing country like Brazil.

**Objectives:**

to characterize the cognitive functioning of healthy elderly subjects whose socioeconomic conditions differ to those of other studies.

**Methods:**

60 elderly subjects with a mean age of 68 years, 43 women and 17 men, and mean schooling of 7.1 years, were studied. The cognitive function of this group was assessed using the following neuropsychological tests: Mattis Dementia Rating Scale (MDRS), Stroop Test, Verbal Fluency, Wisconsin Card Sorting Test (WCST), Rey Complex Figure, Vocabulary – Wais – III, Logical Memory (WMS-R), Visual Reproduction (WMS-R), and Rey Auditory-Verbal Learning Test (RAVLT). The neuropsychological data were submitted to Multivariate cluster analysis using SAS - Proc Cluster software and the complete binding hierarchical method.

**Results:**

Variability was found allowing classification of the studied group into 4 clusters of individuals who had above-average (C1), average (C3 and C4) and below average (C2) performance. Schooling determined the results obtained, with less educated subjects showing poorer performance than higher-educated subjects.

**Conclusions:**

Significant differences in the process of cognitive aging were detected on neuropsychological tests in this group of healthy elderly from the developing country of Brazil, where socioeconomic differences may exacerbate cognitive differences among older adults.

## Abstract (pt)

Com o envelhecimento algumas habilidades cognitivas inevitavelmente declinam. No entanto, as perdas cognitivas não ocorrem da mesma maneira para todos os idosos, diferindo na quantidade e intensidade das funções cognitivas afetadas que podem se intensificar pelas diferenças socioeconômicas encontradas num país em desenvolvimento, como o Brasil.

**Objetivos:**

Caracterizar o funcionamento de idosos saudáveis em condições socioeconômicas diversas das encontradas em outros estudos.

**Métodos:**

60 idosos saudáveis, com idade média 68 anos, 43 mulheres e 17 homens e escolaridade média de 7,1 anos. O funcionamento cognitivo desse grupo foi avaliado por meio da aplicação dos seguintes de testes neuropsicológicos: Escala de Mattis para Avaliação de Demência (MDRS), Teste de Stroop, Fluência Verbal, Teste de Winconsin de Classificação de Cartas- WCST, Figura Complexa de Rey, Vocabulário – Wais – III, Memória Lógica – WMS-R, Reprodução Visual – WMS-R, Teste da Aprendizagem Auditivo-Verbal de Rey. Os dados da avaliação neuropsicológica foram submetidos à análise Multivariada de Cluster pelo *Software* SAS - *Proc Cluster* utilizando o método hierárquico de ligação completa.

**Resultados:**

Demonstraram uma variabilidade que permitiu a classificação do grupo de estudo em 4 clusters de indivíduos que tiveram um melhor desempenho (C1), com desempenho dentro da média (C3 e C4) e com pior desempenho (C2). A escolaridade determinou os resultados encontrados, sendo que os menos escolarizados mostraram piores desempenhos do que os outros.

**Conclusões:**

Houve diferenças significativas no processo de envelhecimento cognitivo estimada pela avaliação neuropsicológica nesse grupo de idosos saudáveis em países em desenvolvimento, como o Brasil, em que as diferenças socioeconômicas podem intensificar as diferenças cognitivas entre os idosos.

With aging, severe cognitive skills such as mental processing rate and memory inevitably decline, whereas skills requiring well learned knowledge and abilities tend to improve and only decay at much more advanced ages.^1^ However, cognitive losses do not occur homogenously in all elderly people and when they do occur tend to differ in number and severity of the affected cognitive functions. Thus, cognitive decline is expected to occur in human development, but is subject to inter- and even intraindividual variations.^1^

Ylikoshi et al.,^2^ Valdois et al.,^3^ Mistrushima, Uchiyama and Satz,^4^ Ritchie et al.,^5^ Gunstad et al.,^6^ Passarino et al.^7^ and Maxson, Berg and Mcclearn^8^ have characterized the heterogeneity of cognitive profiles using the quantitative technique of cluster analysis. This statistical procedure is indicated when the objective is to discover structures in data with no previous explanations or interpretations, so that the association between two objects will be maximal within the same group and minimal outside. In these studies, the hypothesis of variability in cognitive aging was confirmed, with the identification, in general, of groups with above average, average and below average performance based on cluster analysis. Most studies^9-10^ found groups with successful aging, i.e., persons who age under excellent, almost utopical conditions, and others with normal aging, indicating aging free of mental or biological diseases, which is the type most frequently detected in healthy persons. Finally, these studies have observed pathological aging marked by illness and a greater risk of cognitive decline according to the performance in neuropsychological tests.

In developing countries such as Brazil, where life expectancy and the number of elderly persons are increasing, there is the need to study this age range to recognize early pathological conditions. In addition, there is more variability of schooling in our population who is generally less educated than development countries. According to IBGE data obtained in the 2005 National Survey by Residence Sampling in the state of São Paulo there were a predominance of elderly subjects with no schooling or less than 1 year of schooling (32.73%), followed by subjects with 4 to 8 years of schooling (32.5%), subjects with 1 to 3 years of schooling (23.8%), and subjects with 9 years or more (11.1%). Thus, most of these individuals are illiterate or have completed a maximum of 8 years of schooling.

Nitrini et al.^11^ also stated that cognitive evaluation in developing countries is a difficult undertaking due to low levels of schooling and particularly the illiteracy still frequent in the elderly which lead to the study of a more suitable instrument to evaluated illiterate elders. Ostrosky-Solis et al.^12^ found a significant educational effect across different age ranges on most of the neuropsychological tests, although it was more noted in constructional abilities (copying of a figure), language (comprehension), phonological verbal fluency, and conceptual functions (similarities, calculation abilities, and sequences). Even in an epidemiological study of normal aging and dementia in the Northern Manhattan community the literacy status (literate vs. illiterate) had a significant effect on neuropsychological test performance when groups were matched for years of education.^13^

Accompanying the poor education rates it is estimated that 12.4% of the elderly lived on an income of up to ½ a minimum wage, a fact that may be considered a situation of poverty.^14^ It should also be pointed out that, according to the IBGE, the recipients of retirement benefits increased from 76.6% to 84.6% from 1995 to 2005 regarding all individuals aged 65 years or more. Veras^14^ stated that the elderly are in a worse social situation in developing countries after retirement compared to the time when they were working due to a reduction of income. In this respect, the growth of the elderly population in Brazil is associated with poor education rates and an increase in the indicators of poverty.

Ylikoshi et al.,^3^ Valdois et al.,^4^ Mistrushima, Uchiyama and Satz^5^ and Gunstad et al.^6^ detected significant differences between clusters regarding age, whereas in the study by Ylikoshi et al.,^2^ schooling differed only between those who had completed elementary school and those with higher schooling. In other words, in these studies schooling is above the Brazilian indicators and between their participants there were no significant differences for education, representing a more homogeneous socio-educational pattern when compared to the Brazilians.

Thus, the demographic and social indicators for Brazil as a whole and for the state of São Paulo in particular differ from those reported in developed countries and justify investigations about the characterization of cognitive functioning in order to understand what is considered normal in developing countries like Brazil where socioeconomic differences may magnify cognitive gaps among older adults. The aim of the present study is that in countries with more differences in social and educational parameters there should be more heterogeneity in cognitive function in healthy elders. Finally, these arguments bring up a simple question how we would expect to be cognitive function in a different socioeconomic environment, like the Brazilians.

## Patients and methods

### Patients

The series consisted of 60 elderly subjects aged 58 to 83 years (mean±SD: 68.4±6.17), 43 women and 17 men with 1 to 15 years of schooling (7.1±4.39) (Table 1) from community, Clinical Hospital of the School of Medicine from Ribeirão Preto – USP and private office. All subjects were diagnosed as healthy which means without disease that compromise cognitive and cerebral functioning. The study was approved by the Research Ethics Committee of the Clinical Hospital of the Faculty of Medicine of Ribeirão Preto – USP and all subjects gave written informed consent to participate in the study.

**Table 1:** Frequency table of demographic data for the 4 Clusters (C).

Clusters		C1 (n=13)	C2 (n=20)	C3 (n=17)	C4 (n=10)	p
Age (mean)		63.46	74.15	63.18	72.4	<0.01**
Gender	Female	8	11	16	8	<0.04*
	Male	5	9	1	2	
Schooling (years)		12.5	3.3	4.7	11.7	<0.01**
Hand dominance	Dextrous	13	19	16	10	
	Ambidextrous	0	1	0	0	
	Left-handed	0	0	1	0	
Occupation	Retirees	5	9	9	6	0.81*
	Housewives	3	7	3	3	
	Active workers	5	4	5	1	
NSE	Medium	3	1	0	1	<0.01*
	Medium-inferior	8	0	1	5	
	Low-superior	2	11	14	2	
	Low-inferior	0	7	2	1	
	Not classified	0	1	0	0	
Vocabulary (crude scores)		49	34.7	36.06	41.9	<0.01**

* p-value referring to the Chi-square test;

** P-Value referring to one-way analysis of variance;

*** NSE, socioeconomic classification.

### Procedures

A geriatrician (FP) first evaluated all participants and medical conditions that might interfere with cognitive and cerebral functioning. The instruments used to test the patients were the MINI International Neuropsychiatric Interview – Brazilian version^15,16^ and the MEEM.^17^ In addition, laboratory tests were performed (VDRL, HMG, calcium, fasting glycemia and TSH) and the Katz and Clinical Dementia Rating Scale (CDR) indices were determined.^18^ Exclusion criteria were: affections of the central nervous system that compromised cognitive function, subjects with sensorineural deficits or sensorimotor incapacitation that would impair the execution of the proposed tests (important hearing loss, visual or color recognition deficit); individuals with psychiatric disorders, subjects with a history of chronic alcoholism (>3 doses/d), clinical delirium, pulmonary affections (diagnosis of severe (p0_2_< 60) or oxygen-dependent, pulmonary disease and severe refractory asthma), endocrine-metabolic and nutritional affections determined by laboratory tests such as VDRL, calcium, fasting glycemia, HMG, and TSH, i.e., diabetes mellitus, hypo/hyperthyroidism, hypercalcemia, GH deficiency, conditions of hypo- or hypercortisolism, vitamin B12. folic acid and niacin deficiency, and anemia with hemoglobin below 10 mg/dl; cardiovascular disorders; a diagnosis of severe or end-stage heart disease, a history of acute myocardial infarction or documented coronary disease, moderate to severe hypertension, advanced atherosclerosis; use of the following medications: neuroleptics, tricyclic antidepressants, anticonvulsants, methyldopa, clonidine or similar drugs, corticoids (>5 mg prednisone or equivalent), and benzodiazepines < 6 months or >10 mg diazepam or equivalent.

Healthy individuals were submitted first to socioeconomic classification^19^ and then to the neuropsychological evaluation using Mattis Dementia Rating Scale (MDRS),^20-21^ Stroop Test, ^21-22^ Verbal Fluency,^23^ Wisconsin Card Sorting Test (WCST),^24-25^ Rey Complex Figure,^26^ Vocabulary – Wais – III,^27^ Logical Memory (WMS-R),^28-29^ Visual Reproduction (WMS-R),^30-31^ and Rey Auditory-Verbal Learning Test (RAVLT)^32^ conducted by a neuropsychology with experience in aging. Other demographic variables were collected by informal questionnaire with the patient such as schooling, occupation, socioeconomic level and manual dexterity.

### Data analysis

The performance of each participant on the neuropsychological evaluation was represented by the following indices: total MDRS; RAVLT: A1 to A5 total, A after 30 minutes, and Recognition; WMS-R: Logical Memory I and % retention of logical memory, Visual Reproduction and % retention of Visual Reproduction; Verbal Fluency (animals); Rey Complex Figure: copy and evocation after 5 minutes; Stroop interference index, and WCST: errors, perseverative responses, perseverative errors and categories. The data were first evaluated to verify the colinearity and after submitted to Multivariate Cluster Analysis using the Proc Cluster SAS software and the complete linkage hierarchical method.

Exploratory analyses were also carried out on the demographic data distributed in the clusters using one-way ANOVA or the χ^2^ test of the SPSS 13.0 software for Windows.

## Results

All 16 variables selected were included in the cluster analysis since all presented interdependence after testing for co-linearity. The variables were standardized as z- scores according to the mean and standard deviation of this study group, and signs were inverted for those measures in which a higher score indicated a poorer performance, as was the case for the measures of processing time.

Following application of the statistical procedures, cluster analysis revealed four clusters. The demographic data of the clusters (Table 1) showed a division between older (72 to 74 years) and younger (63 years) elderly subjects. The same occurred with schooling which yielded two groups, one containing individuals with incomplete elementary school education (up to 5 years) and another with those who had high school education (12 years). Schooling is known to unify and interact with other demographic variables to some degree, and accordingly our less educated elderly subjects had poorer performance on the Vocabulary–Wais III than did more educated individuals, possibly because they did not have the opportunity to increase their lexical and semantic vocabulary through a longer period in school. In addition, their occupations were not specialized, as was the case for lower socioeconomic levels observed in Cluster 2. This coincides with the sociodemographic indicators of the present study in which schooling, occupation and socioeconomic level were inter-related and appeared to reflect the reality in Brazil among retired elderly.

The clusters also differed regarding male and female ratio where Clusters 3 and 4 contained a larger number of women than men, suggesting an influence of gender on these results. There was a prevalence of dominance of right handedness, leading us to believe that the left hemisphere was dominant for language in most of the subjects in our series.

The stability and reliability of this cluster formation were tested by removing the 16 variables one by one, and applying cluster analysis again. The results showed that the characteristics of these groups continued to be similar, while the mean values of the neuropsychological variables showed no significant changes.

External validation was carried out by a one-way ANOVA in which the clusters represented the independent variable, and other neuropsychological indices (MDRS attention, MDRS initiation/perseveration, MDRS construction, MDRS conceptualization, MDRS memory, M Logic II, R Visual II, Ravlt V, Total Vocabulary l, Fluency FAS, Stroop D, and Stroop C) represented the 12 dependent variables. This analysis revealed a statistically significant difference (p< 0.05) among these variables thereby confirming the validity of this cluster formation.

**Figure 1:**
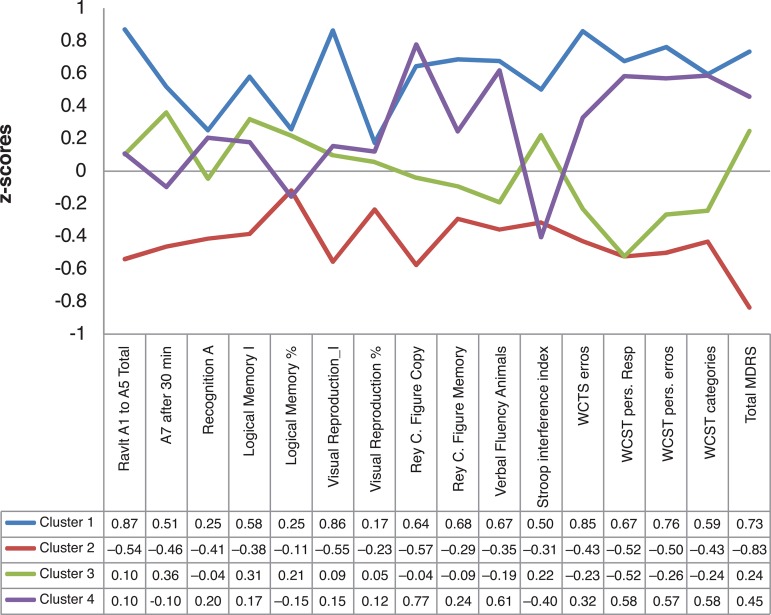
Comparison of z-scores by cluster.

Thus, this cluster solution proved to be stable, reliable and valid, permitting the continuity of analysis, with the characterization and classification of the groups. Cluster 1 (n=13) represented a group of younger elderly subjects, with greater schooling and higher socioeconomic level, including a larger number of active workers with indicators of greater preservation of previous cognitive functioning compared to the remaining groups (Table 1). This cluster was characterized by a majority of right-handed subjects and a mean value of crude weighted scores which ranged from +0.869 to +0.173, and above average performance (Figure 1). In contrast, Cluster 2 (n=20) was composed of older subjects with lower schooling and socioeconomic level and contained a larger number of retirees. This cluster comprised 19 right-handed subjects, and had a mean performance which ranged from –0.837 to –0.1191, characterizing a group with more negative scores, i.e., below average and worse than the other clusters (Figure 1).

Cluster 3 (n=17) was similar to Cluster 2 in terms of lower schooling and lower socioeconomic level, although poverty indicators were better and subjects were younger. One of the main characteristics of this group compared to the others was the disproportionally higher number of women (Table 1). The group also comprised a majority of right-handed subjects and its neuropsychological profile indicated average performance with z-scores ranging from –0.266 to +0.359, (Figure 1) and worst performance in mental flexibility related to executive functioning.

**Figure 2:**
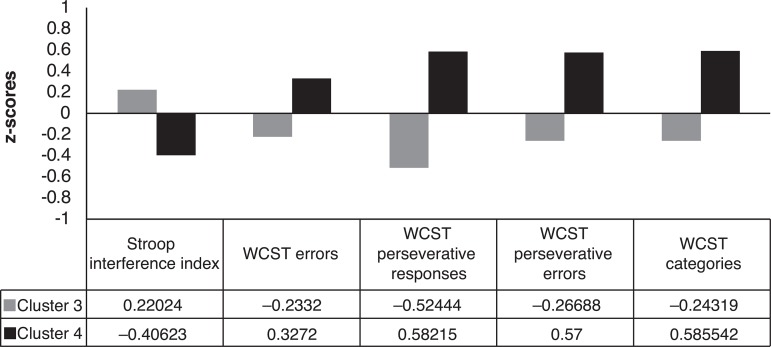
Difference in cognitive profiles between Clusters 3 and 4.

Cluster 4 (n=10) had similar schooling to Cluster 1, but with more indicators of poverty and a larger number of retirees. Also, Cluster 4 subjects were older than those in Cluster 1, where this was associated with more retired subjects and lower socioeconomic level. All participants were right-handed and the results of the neuropsychological tests revealed average performance, with mean scores ranging from +0.778 to –0.406, while individuals had greater difficulty in inhibitory control of interference on the Stroop test (Figure 2).

None of the clusters presented performance indicative diagnosis of dementia according to DSM-IV. However, in general, the performance of the groups was such that Cluster 1 showed no negative performance, whereas Cluster 2 only presented negative results, i.e., below the mean.

Clusters 3 and 4 both presented average performance (Z-score=0), although the frequency of negative performances was lower in Cluster 4 (Figure 1), considering that Cluster 3 also had lower schooling than Cluster 4.

Differences between the groups were also found in terms of the profiles of cognitive skills and difficulties presented, especially between Clusters 3 and 4. These groupings differed from one another in performance on the WCST and Stroop test, with Cluster 3 presenting worse performance on the WCST and preserved performance on the Stroop test, whereas Cluster 4 revealed greatest difficulties on the Stroop test, with preservation on the WCST (Figure 2).

In Cluster 4, there was a fall in performance between immediate and delayed recall, demonstrated on the RAVLT (learning trial and evocation after 30 minutes) and the Logical Memory I subtest – WMS-R (immediate evocation and after 30 minutes of the Logical Memory) (Figure 1). This decline was also evident on the recall of the Rey Complex Figure after 3 minutes, and the copy that was intact.

## Discussion

Cognitive aging has been found to be heterogeneous in other studies and the issue that should now be addressed is the characterization of cognition among an elderly population with diverse socioeconomic conditions. In parallel, such an investigation might also provide a profile of normal performance in this study group. To this end, the present study analyzed performance on neuropsychological tests using the exploratory method and cluster analysis, given the profusion of results and lack of consensus regarding the parameters of cognitive aging.

According to this statistical criterion and considering the clinical significance of the data, we initially reached a solution with 4 clusters, which proved to be stable, reliable and valid for the present study. The clusters showed a division between age and schooling, a finding not observed in developed countries, which tend to have higher schooling levels and no significant differences between clusters regarding educational level.^2-8^

Other authours analysed the influence of age and education on neuropsychological tests and identified the higher effect of education though age, since they compared a group with a limited education (0-4 anos) and a wide age range (16-85 years) and still found a preponderance of the education effect^12^. Nitrini et al.^11^ evaluated a group of healthy elders and described differences in in memory performance (delayed recall) between literates and illiterates ones. Also differences in working memory were verified in elders according to education level, associating a better working memory with higher levels of schooling.^33^ Differences in organization of visuoespatial information, lack of previous exposure to stimuli, and difficulties with interpretation of the logical functions of language could be possible explanations for the discrepancies between literates and illiterates.^13^

Schooling and age can also be considered risk factors for Alzheimer’s disease.^34^ Meguro et al.^35^ also found a significant correlation between age and atrophy of the frontal lobe in a group with low schooling. In fact, schooling seemed to be a protective factor for cognitive decline associated with aging, probably because it increase the number of synapses and vascularization of the brain, i.e., the hypothesis of cognitive reserve.

Baltes and Baltes postulated that cognitive aging was not the same in all individuals, having persons who exhibit successful cognitive aging, normal aging or pathological aging. According to these investigators, Cluster 1 could represent the group with “successful” cognitive aging, with all results above the mean and greater schooling and younger age than the other clusters. Clusters 3 and 4 presented performances within the mean and therefore should be classified as having normal cognitive aging, while Cluster 2 could be classified as the group with “pathological” cognitive aging and at risk for developing neurodegenerative diseases, since all the scores were below the mean and individuals had the lowest educational levels. However, these tendencies need future confirmation by a longitudinal study and use of normative data for these neuropsychological parameters.

It should be emphasized that none of the clusters presented performance indicative of diagnosis of dementia according to DSM-IV. It should also be pointed out that all participants were evaluated and selected based on clinical and laboratory tests by a geriatrician specifically trained in diagnosing dementia, who referred the subjects for neuropsychological evaluation and MRI. Thus, subclinical diseases that might explain cognitive alterations were excluded, with only healthy elderly subjects being selected.

Differences between clusters also involve qualitative aspects of cognitive profiles, observed mainly between Cluster 3 and Cluster 4, both of which had average performances. The performance of Cluster 3 was average for almost all techniques applied, except for WCST which showed a decrease in perseverative responses compared to other indices which remained more within the mean. This fact may be explained by the mean schooling of 4.7 years in this cluster, given that the WCST is influenced by educational level. Consequently, the larger number of perseverative responses in Cluster 3 may reveal an inability to switch from one category to another, or to verify new strategies of reaching certain objectives, where this deficit is related to low schooling and possibly to lack of development of these skills.

In the Stroop Test, however, schooling was not sufficient to explain the performance of Cluster 4, which only required automation of reading, a skill already acquired and consolidated at the educational level of this group. Wetter et al. (2005) observed that individuals with APOE ε4 committed a larger number of errors on the D-KES Color-Word Interference Test, a variation of the Stroop test containing an additional condition of inhibition and switching, than individuals without APOE ε4. This also applies to the interference rate, in which the greater slowness between the interference condition and baseline suggests losses of inhibitory control, also detected in the early phases of Alzheimer’s disease. Thus, age may be a factor that affected this performance because the subjects in Cluster 4 were older than those in Cluster 3.

Cluster 4 also presented losses in delayed recall trials or in percent retention, with preservation of learning or of immediate recall, i.e., a preponderance of losses in retention among the mnesic processes evaluated, although all of these performances were practically at the level of the mean for this group. This cognitive profile, together with the changes in the effect of interference, suggests the possibility that Cluster 4 is at risk for developing future diseases that are compensated by the high schooling of the group.

We conclude that, based on neuropsychological tests, there is variability in the cognitive performance of elderly subjects. Schooling and socioeconomic conditions influenced the results obtained, with less educated subjects showing a poorer performance than subjects with greater schooling. Studies of this type are also important to improve the understanding of minimal cognitive dysfunction included in the concept of normal aging in developing countries like Brazil. A limitation to the discussion of this study is the lack of normative data for the neuropsychological tests used, where this hampered identifying of impaired ranges and precludeds comparison with other groups. It should also be noted that the present results are preliminary and should be extended through future studies.
